# The study of the removal of penconazole fungicide from surface water using carboxymethyl tragacanth-based hydrogel grafted with poly (acrylic acid-*co*-acrylamide)

**DOI:** 10.1038/s41598-023-40862-7

**Published:** 2023-08-21

**Authors:** Magsoud Lotfi, Morteza Bahram, Peyman Najafi Moghadam

**Affiliations:** 1 Department of Analytical Chemistry, Faculty of Chemistry, Urmia University, Urmia, Iran; 2 Department of Organic Chemistry, Faculty of Chemistry, Urmia University, Urmia, Iran

**Keywords:** Analytical chemistry, Polymer chemistry, Chemical synthesis

## Abstract

In this study, a polymeric adsorbent based on carboxymethyl tragacanth (CMT) grafted by poly acrylic acid-*co*-acrylamide (AAc-*co*-AAm) synthesized by radical polymerization for the first time was used to remove the fungicide penconazole (PEN) or Topas 20% from surface water. The parameters of solution pH, adsorption isotherm, and adsorption kinetics of PEN were studied by the synthetic adsorbent. The surface morphology and functional groups of CMT-g-poly (AAc-*co*-AAm) were confirmed by XRD, SEM and FT-IR techniques. Adsorption of PEN on CMT-g-poly (AAc-*co*-AAm) follows the Freundlich and pseudo-second-order models. The significant maximum adsorption capacity of the synthesized polymer was found to be 196.08 mg/g. The synthetic adsorbent had good reproducibility in PEN removal for up to 5 cycles. CMT-g-poly (AAc-*co*-AAm) is a cost-effective and non-toxic adsorbent for the decontamination of surface water from pesticides.

## Introduction

**Figure 1:**
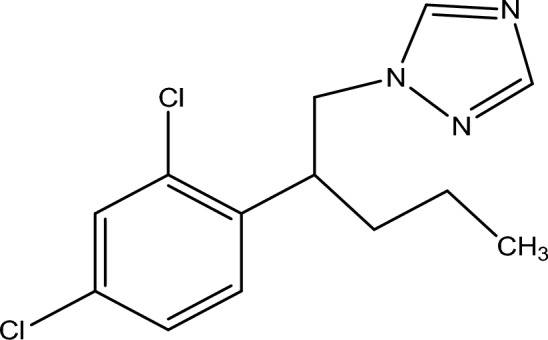
Chemical structure of penconazole.

**Figure 2:**
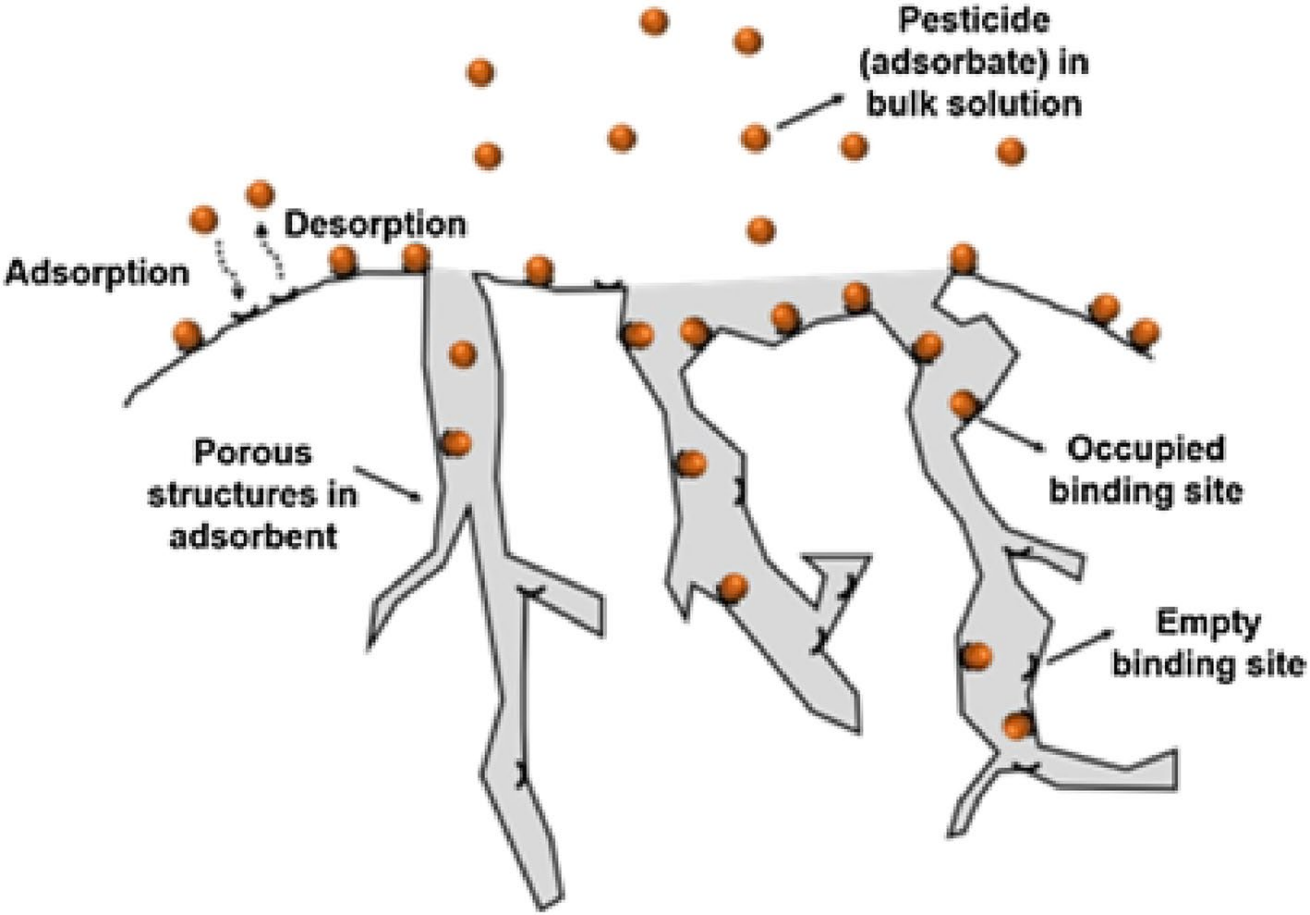
Surface adsorption process.

Water is the most valuable and important substance needed by humans and its use and importance are shown in drinking, sanitary, agricultural, and industrial uses. The ever-increasing growth of the population, the improvement of the standard of living, and the development of urbanization are among the factors that cause an increase in water consumption and wastewater production and cause environmental pollution^1^. Meanwhile, pesticides, which are emerging contaminant, are among the most important and widely used poisons that are used in agriculture as insecticides to kill insects and arthropods, or as herbicides to fight weeds. The use of new technology in agriculture has led to the use of these materials to harvest more crops, however, the excessive use of pesticides has caused soil pollution and the entry of large amounts of these toxins into water sources^2^. Although fungicides have received less attention compared to other agricultural toxins, fungal diseases are considered a great threat to agricultural products. Fungicides are toxic to a wide range of organisms and are dangerous to aquatic life. Fungicides, like pyrethroid and organophosphate insecticides, are lipophilic. The effect of fungicides in the environment takes place directly or indirectly through another species that is affected by them^3^. Triazole fungicides are a group of heterocyclic compounds that have at least one five-membered ring of two carbon atoms and three nitrogen atoms and are widely used to prevent and treat various fungal diseases in agricultural products. These fungicides currently constitute 25 commercial agrochemicals worldwide. Also, they have good penetration and durability in soil and water, and their high consumption causes side effects related to endocrine glands in humans and animals^4^. PEN or (R, S)-1-[2-(2, 4-dichlorophenyl) pentyl]-1*H*-1, 2, 4-triazole is a fungicide from the triazole family that is used to control powdery mildew and other pathogenic ascomycetes, basidiomycetes, and Deuteromycetes. The chemical structure of PEN is shown in Fig. 1. This fungicide is marketed under the brand name Topas 20% and is classified by the European Food Safety Organization as a dangerous substance for humans and the environment, so finding a way to remove it from the environment is very important^5^. In a study conducted by Nicoleta et al. to remove PEN from water using montmorillonite clay, the adsorption capacity of 6.33 mg/g of PEN by montmorillonite was obtained^6^. Considering that the studies conducted in the field of removal of PEN by surface adsorption method are limited, therefore we decided to use this method in the recent study. By using physical, chemical, and biological methods, pesticides can be removed from water sources. Membrane and adsorption processes are among the physical methods that rely on separation. The challenge of membrane fouling is the main limitation concerning the removal of pesticides with this method because it disrupts the performance of the membrane in separation^7^. The surface adsorption method on solid and porous substrates has a significant advantage over other water and wastewater treatment techniques due to its low cost, ease of use, production of fewer byproducts, and easy integration with other techniques for better efficiency^8^. The principles of the surface adsorption process are shown in Fig. 2. The mass transfer takes place when the solution contaminated with the pollutant comes into contact with the adsorbent. Pollutant-adsorbed species are selectively transported from the bulk solution and occupy the binding sites on the surface of the adsorbent. Based on the nature of the interaction between the adsorbent and the adsorbate, the phenomenon of surface adsorption is chemical or physical. Physical surface adsorption leads to the adsorption of several layers of contaminant on the adsorbent and is also endothermic and reversible, while chemical surface adsorption is a dense single-layer adsorption and is also an exothermic and irreversible process and is stronger than physical adsorption^9^. Activated carbon, Biochar, montmorillonite clay, and hydrogels are the most important adsorbents used to remove pesticides from water and wastewater^10–12^. Hydrogel is a swellable, hydrophilic, and insoluble three-dimensional polymer network that is produced by the reaction of one or more monomers. These features distinguish hydrogels from other polymers. Based on the origin of the polymer, hydrogels are divided into two categories: natural and synthetic. Natural hydrogels have a long life, high water adsorption capacity, and high gel strength and have gradually replaced synthetic hydrogels^13^. Tragacanth Gum (TG) is one of the inexpensive natural polysaccharides that is obtained from the dried sap of the Astragalus, and due to the presence of hydroxyl, carboxylic, and epoxy functional groups, it can be used in the polymerization reaction with various reagents. This gum is non-toxic, biocompatible, and stable in a wide range of pH^14^. Hydrogels based on acrylic acid, due to high swelling, high adsorption capacity, and high adsorption speed, have found wide applications in the field of preparation of polymer absorbents for water and wastewater treatment. The most common synthesis method for these hydrogels is the radical polymerization method. The degree of crosslinking is one of the most important factors in the adsorption capacity of these hydrogels. Various adsorption mechanisms have been proposed by these hydrogels, such as electrostatic interactions, hydrophobic interactions, ion exchange, and hydrogen bonding^15^. Also, acrylamide-based hydrogels are the most commonly used hydrogels and show a significant volume change in response to physical and chemical stimuli. These hydrogels are used to remove various contaminant^16,17^. In this study, for the first time, we used carboxymethyl tragacanth-based hydrogel grafted with poly (acrylic acid-*co*-acrylamide) to remove PEN from aqueous solutions and parameters of adsorbent contact time with the contaminant, contaminant concentration, and solution pH were evaluated.

## Material and methods

### Materials

In this study, tragacanth gum (TG) of commercial grade was purchased from a medicinal plant shop in Tabriz, Iran. The acrylamide (AAm), acrylic acid (AAc), *N*,*N*′-methylene bisacrylamide (MBA), and ammonium peroxydisulfate (APS) were purchased from Merck. To prepare the PEN solution, Topas 20% commercial fungicide was used. The isopropyl alcohol (C_3_H_8_O), chloroacetic acid (MCA), and sodium hydroxide (NaOH) were analytical grades and were used without further purification.

### Synthesis of carboxymethyl tragacanth (CMT)

Carboxymethylation was carried out for the functionalization of the tragacanth surface by carboxyl acid. Also the carboxyl groups next to hydroxyl groups are the sites of polymerization for radical graft polymerization. So in a 100 mL round bottom flask equipped with a magnetic stirring bar and a reflux condenser, 2 g of tragacanth were stirred in a 44 mL isopropanol/deionized water solution with a ratio of 31/13. Then, 2.4 g of sodium hydroxide were added to the contents of the flask and the reaction mixture was heated to 60 °C for 30 min. Next, 3 g of monochloroacetic acid were added to it, then the temperature of the reaction mixture was increased to 70 °C and stirred for another 4 h. After completion of the synthesis reaction, the reaction mixture was allowed to cool to room temperature, and the organic solvent was removed by evaporation. Then the aqueous phase was neutralized by acetic acid. Cold methanol was added to the reaction mixture as an anti-solvent, and the collected precipitate was washed with methanol and dried in a vacuum, and finally the CMT was obtained.

### Synthesis of CMT-g-poly (AAc-*co*-AAm) copolymer

**Figure 3:**
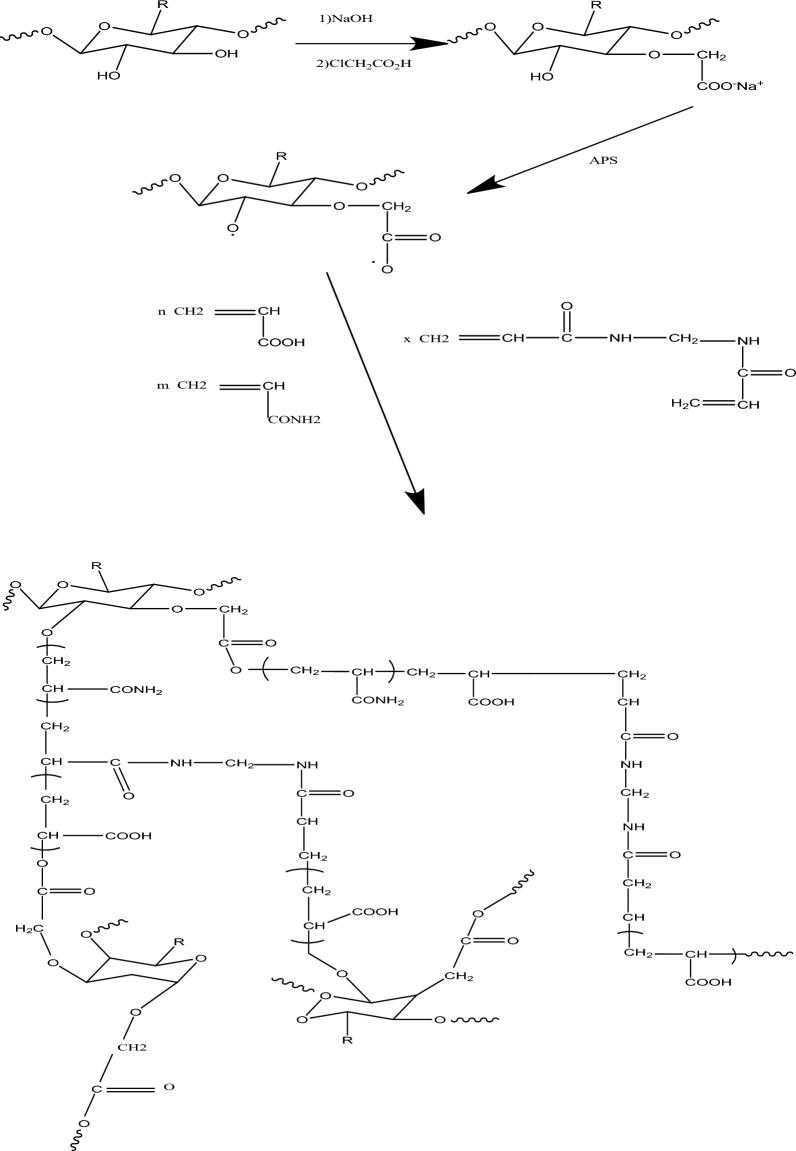
Mechanism of formation CMT-g-poly (AAc-*co*-AAm) copolymer.

For the main step of hydrogel synthesis, in a round-bottomed flask equipped with a magnetic stirrer and a reflux condenser, 0.6 g of powdered CMT synthesized in the previous step was dissolved in 100 mL of deionized water. Then the solution was deoxygenated by bubbling argon gas for 20 min. Next, 0.057 g of APS was added to activate the polymerization sites in carboxymethyl tragacanth and to start the graft polymerization, and the reaction mixture was heated to a temperature of 50 °C for 10 min. After that, 0.385 g of MBA, 1.8 g of acrylic acid, and 1.8 g of acrylamide were added and after a few minutes, a gel was formed in the reaction mixture. Figure 3 show the mechanism of formation CMT-g-poly (AAc-*co*-AAm) copolymer. The obtained gel was separated and washed with a methanol/water mixture and dried in a vacuum oven to constant weight. The obtained CMT-g-poly (AAc-*co*-AAm) copolymer is used to investigate the removal of PEN from an aqueous medium^18^. Compared to the previous study, acrylamide monomer has been used in the synthesis of hydrogel. This monomer is widely used in the synthesis of hydrogels^19^. The use of acrylamide and acrylic acid monomers in graft copolymerization has a great effect for adjusting the hydrophilicity of hydrogel and also the synthesis of hydrogel with high ability in hydrogen bond formation and good interaction with penconazole fungicide molecules. The objective of this study is the possibility of using this polymeric adsorbent in the removal of pesticides, as one of the main species of water pollution.

### Characterization

Fourier transform infrared (FT-IR) spectra for TG, CMT and CMT-g-poly (AAm-*co*-AAc) were acquired by the KBr disc method using a Thermo-AVATAR FT-IR spectrophotometer. Scanning electron microscopy (SEM) was used for the morphological elucidation of TG, CMT and CMT-g-poly (AAc-*co*-AAm) using the FE-SEM model ZEISS Sigma 300. Also, the x-ray diffraction (XRD) spectra for TG, CMT, and CMT-g-poly (AAm-*co*-AAc) were obtained using the XRD model Rigaku Ultima IV.

### Preparation of PEN solution

The stock solution of PEN of 100 mg L^−1^ concentration was prepared using a 20% commercial Topaz solution in a 100 mL balloon with a 4:1 ratio of deionized water and acetonitrile. Working solutions were prepared for each experiment by diluting the above solution. All UV–Vis spectra of PEN were recorded using an Agilent 8453 spectrophotometer equipped with a diode array detector in a 1 cm pathlength quartz cuvette. The standard calibration curve for PEN was also determined using PEN solutions of various concentrations.

### Measuring the swelling of CMT-g-poly (AAc-*co*-AAm)

At first, 0.01 g of dry CMT-g-poly (AAc-*co*-AAm) was placed in water, and after different periods, the water was drained and the weight of the swollen CMT-g-poly (AAc-*co*-AAm) was measured. Water absorption inside the hydrogel matrix is determined by the difference between the weight of the swollen CMT-g-poly (AAc-*co*-AAm) and the dry CMT-g-poly (AAc-*co*-AAm) according to Eq. (1)^20^.

1 $$Water uptake=\frac{Wwet-Wdry}{Wdry}\times 100$$

where W_wet_ is the weight of the swollen CMT-g-poly (AAc-*co*-AAm) and W_dry_ is the weight of the dry CMT-g-poly (AAc-*co*-AAm). Next, the swelling rate of the synthetic hydrogel was investigated in acidic and alkaline media.

### Adsorption of PEN on CMT-g-poly (AAc-*co*-AAm)

Adsorption of PEN on CMT-g-poly (AAc-*co*-AAm) was studied by batch experiments. For this purpose, 30 mL PEN solution (30 mg L^−1^) was mixed with 10 mg CMT-g-poly (AAm-*co*-AAc) and shaken at 120 rpm at room temperature for a certain time. All samples were centrifuged and analyzed by UV–Vis spectrophotometer to ensure the residual concentration of PEN in the solution. The concentration of PEN before and after adsorption was measured using the calibration curve of PEN. Finally, the amount of PEN absorbed by the adsorbent was calculated using Eq. (2)^21^.

2 $${q}_{e}=\frac{\left({C}_{0}-{C}_{e}\right)\times V}{M}$$

In this equation, q_e_ (mg g^−1^) is the adsorption rate of the adsorbate at equilibrium, C_0_ (mg L^−1^) is the initial concentration of PEN, C_e_ (mg L^−1^) is the equilibrium concentration of PEN after contact with the adsorbent, M (g) is the amount of the adsorbent used and V (L) is the sample volume.

### Investigation of adsorption kinetics

For adsorption kinetic experiments, 30 mL PEN solution (30 mg L^−1^) was mixed with 10 mg CMT-g-poly (AAm-*co*-AAc) and shaken at 120 rpm at room temperature for specific time intervals. Laboratory data were fitted with pseudo-first-order (PFO), pseudo-second-order (PSO), and Elovich kinetic models. The nonlinear form of the PFO model is shown in Eq. (3)^22^.

3 $${q}_{t}={q}_{e}\left(1-{e}^{{-k}_{1}t}\right)$$

In this equation q_e_ (mg g^−1^) and q_t_ (mg g^−1^) are PEN adsorbed on CMT-g-poly (AAm-*co*-AAc) at equilibrium time and time t (min), and k_1_ (min^−1^) is the constant of the PFO model. Also, the experimental data was evaluated by the PSO model, which is given in Eq. (4)^22^.

4 $${q}_{t}= \frac{{k}_{2}{q}_{e}^{2}t}{1+{k}_{2}{q}_{e}t}$$

In this equation, K_2_ (g mg^−1^ min^−1^) is the speed constant of the PSO model^22^.

Elovich kinetic model is also expressed by Eq. (5) that in this equation, α (mg g^−1^ min^−1^) is the initial adsorption rate, and β (g mg^−1^) is the desorption constant^22^.

5 $${q}_{t}=\frac{1}{\beta }\mathrm{ln}\left(1+\alpha \beta t\right)$$

### Adsorption isotherm studies

Adsorption isotherm studies of PEN by CMT-g-poly (AAm-*co*-AAc) were carried out by mixing 10 mg of CMT-g-poly (AAc-*co*-AAm) with 30 mL of different concentrations of PEN solutions in Falcon tubes. These tubes were shaken at 120 rpm at room temperature for 45 min. The samples were centrifuged and the remaining concentration of PEN was determined by a UV–Vis spectrophotometer. To investigate the equilibrium adsorption isotherms of PEN on CMT-g-poly (AAc-*co*-AAm), three Langmuir, Freundlich, and Temkin models were studied. The Langmuir model, which is based on the assumption of monolayer adsorption of the pollutant and also the reversibility of surface adsorption, is presented by Eq. (6)^23^.

6 $${q}_{e}=\frac{{q}_{max}{K}_{L}{C}_{e}}{1+{K}_{L}{C}_{e}}$$

In this equation, q_e_ (mg g^−1^) is the amount of adsorption per unit surface of the adsorbent at the equilibrium time, C_e_ (mg L^−1^) is the equilibrium concentration in the solution, q_max_ (mg g^−1^) is the maximum adsorption capacity of PEN, and K_L_ (L mg^−1^) is the Langmuir adsorption equilibrium constant, which depends on the adsorption energy. The next equation that was investigated to investigate the isotherm of the surface adsorption process of PEN is the Freundlich model, which is shown in Eq. (7)^23^.

7 $${q}_{e}={K}_{F}{C}_{e}^\frac{1}{n}$$

In this equation, K_F_ is the adsorption equilibrium constant and n is the energy term, which is a function of the covered surface.

Next, the Temkin isotherm model was also examined, as shown in Eq. (8)^24^.

8 $${q}_{e}={B}_{T}ln{(A}_{T}{C}_{e})$$

where A_T_ (L g^−1^) and B_T_ are the Temkin isotherm model constants.

### Regeneration PEN-adsorbed hydrogel

To investigate the reproducibility of CMT-g-poly (AAc-*co*-AAm) in the removal of PEN, a certain amount (20 mg) of adsorbent was placed in contact with 30 mL of 30 mgL^−1^ PEN solution and shaken for 45 min, and after washing and drying the absorbent with 25 mL of 0.1 molL^−1^ NaCl solution, it is placed in a fresh solution of PEN for the next cycle. In the present study, this work was investigated up to 5 cycles and in the pH range of surface waters.

Complying with relevant institutional, national, and international guidelines and legislation. The authors declare that all relevant institutional, national, and international guidelines and legislation were respected.

## Results and discussion

### Polymer characterization

**Figure 4:**
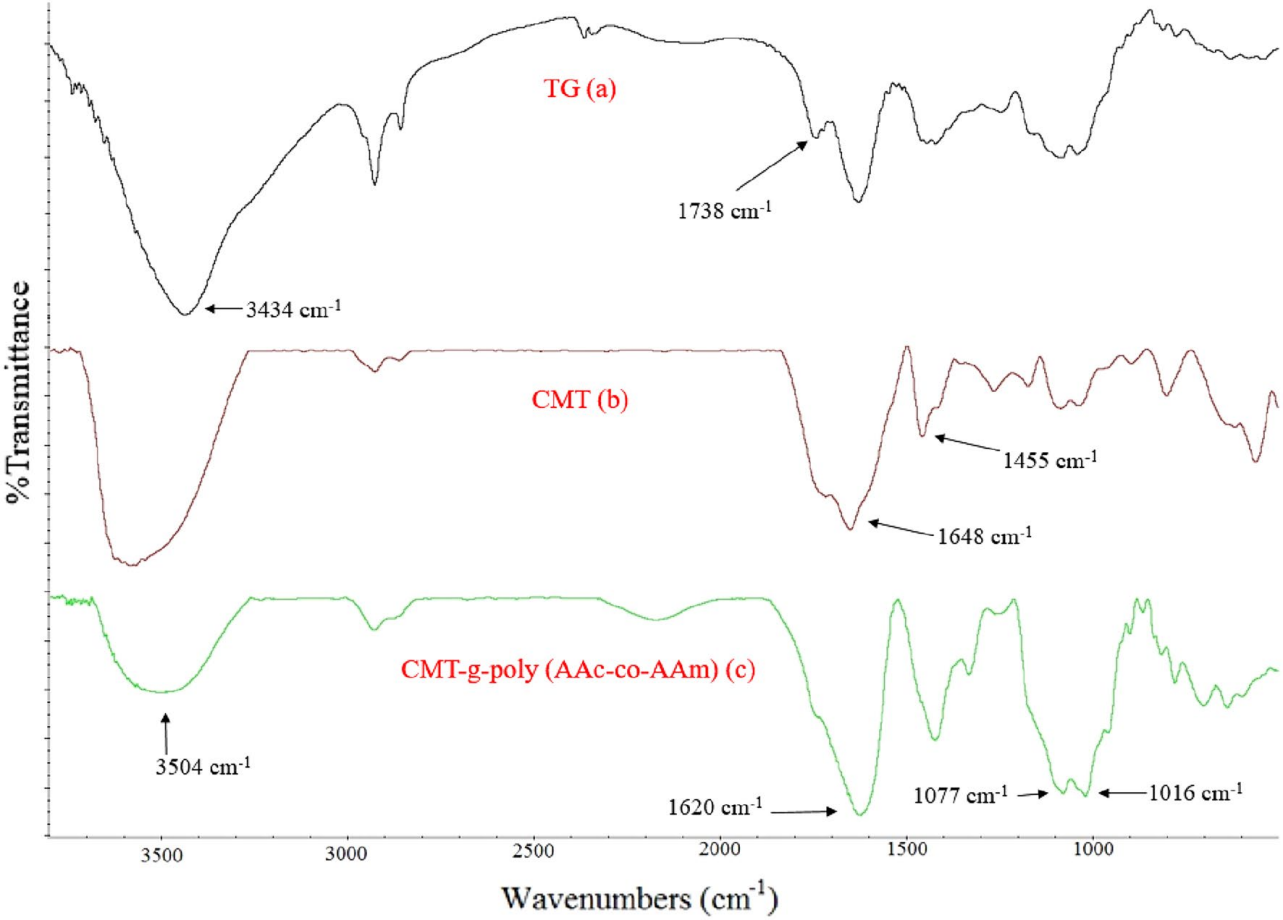
The FT-IR spectra of TG (**a**), CMT (**b**) and CMT-g-poly (AAc-*co*-AAm) (**c**).

The FT-IR spectra of TG, CMT, and CMT-g-poly (AAc-*co*-AAm) have been shown in Fig. 4a–c. The broad peak in the region of 3434 cm^−1^ in Fig. 4a is related to the stretching vibrations of the OH groups in the TG. The absorption peak in 1738 cm^−1^ is assigned to the esteric carbonyl groups in the TG. In Fig. 4b, the absorption peak at 1455 cm^−1^ corresponds to the bending vibration of –CH_2_ of CMT. The absorption peak 1648 cm^−1^ is related to the asymmetric stretching vibration –COO–, which overlaps with the acidic carbonyl groups and ester units of CMT and creates a broad peak. According to Fig. 4c the absorption peak observed at 3504 cm^−1^ is related to the acidic hydroxyl groups of AAc, which overlaps with the N–H stretching vibrations of AAm. The absorption peak 1600–1700 cm^−1^ is intensified compared to the adsorption peak of the carboxymethyl carbonyl group due to the carbonyl groups of AAc and AAm in the graft copolymer. The absorption peaks of 1077 cm^−1^ and 1016 cm^−1^ indicate the stretching vibration of C–O, which is created in the formation of a copolymer. These observations can be good evidence for the formation of linkage polymerization^25,26^.

**Figure 5:**
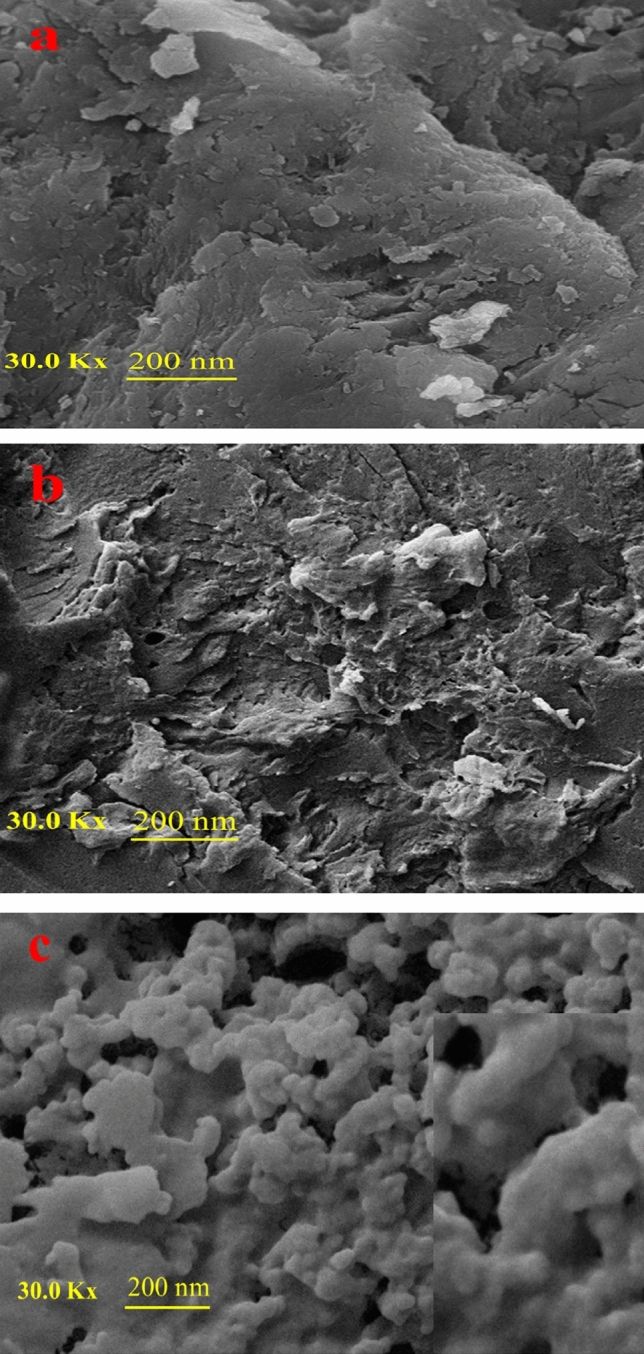
FESEM micrographs of (**a**) TG, (**b**) CMT, and (**c**) CMT-g-poly (AAc-*co*-AAm).

Figure 5 shows the field emission scanning electron microscopy (FESEM) of TG, CMT, and CMT-g-poly (AAc-*co*-AAm). As shown in Fig. 5, the morphology change of the TG (Fig. 5a) surface after its modification is observed in the CMT FESEM image (Fig. 5b). In addition, the rough surface of CMT after graft copolymerization has become softer (Fig. 5c). These images show the uniform growth of poly (AAc-*co*-AAm) on the CMT surface and explicitly confirm the copolymerization of the AAc and AAm on the CMT.

**Figure 6:**
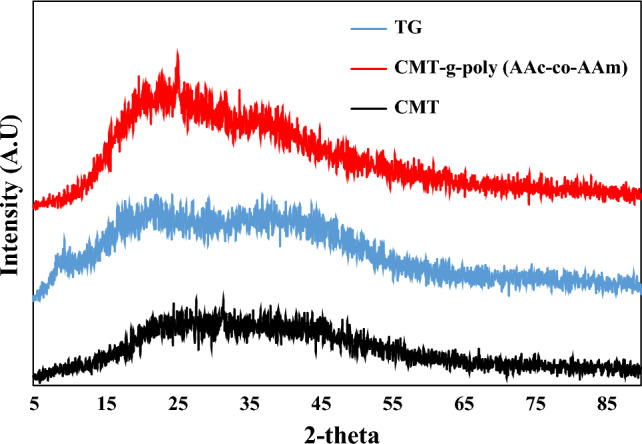
XRD spectra of TG, CMT and CMT-g-poly (AAc-*co*-AAm).

Figure 6 shows the XRD spectra of TG, CMT, and CMT-g-poly (AAc-*co*-AAm). According to Fig. 6, it is clear that TG has partial crystallinity, and after modification and conversion to CMT, there is a clear decrease in crystallinity. The decrease in crystallinity can be attributed to the effect of replacing hydroxyl groups with carboxymethyl groups. Breaking hydrogen bonds leads to a decrease in the crystallinity of TG. After grafting AAc and AAm on CMT, the crystallinity increases due to the increase of hydroxyl groups in CMT-g-poly (AAc-*co*-AAm).

### Swelling of hydrogel

**Figure 7:**
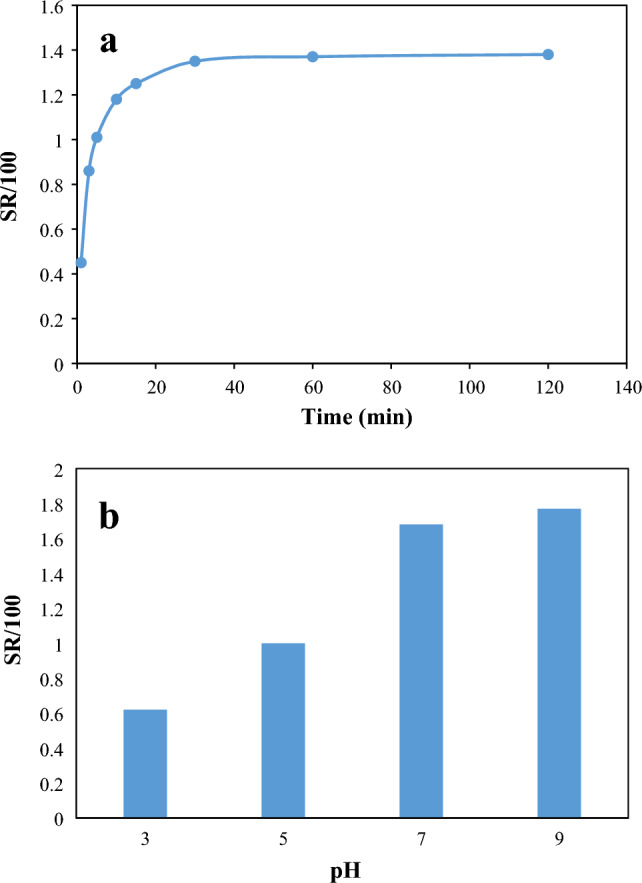
swelling behavior of CMT-g-poly (AAc-*co*-AAm) at (**a**) different time periods and (**b**) different pH.

The higher the swelling rate of the hydrogel absorbent, the higher the adsorption rate and pollutant adsorption rate. To measure CMT-g-poly (AAc-*co*-AAm) swelling, 0.01 g of it was weighed and placed in a container containing 50 mL of deionized water at different periods. Figure 7a shows the swelling ratio (SR, g/g) of the CMT-g-poly (AAc-*co*-AAm) at different periods. As shown in Fig. 7a, the synthetic hydrogel reaches swelling equilibrium after 30 min. In the next step, to check the swelling of the polymer at different pHs, 0.01 g of it was carefully weighed and placed in a beaker containing 50 mL of deionized water with different pHs for 30 min, which is shown in Fig. 7b. According to Fig. 7b, with the increase in the pH of the solution, the amount of CMT-g-poly (AAc-*co*-AAm) swelling increases so that at pH = 9, the SR of the synthetic hydrogel becomes 177 times its initial value. As the pH of the solution increases, the carboxylic groups of the adsorbent are converted into carboxylate anions, and due to the repulsion between the COO^−^ groups, the swelling of the adsorbent in water increases.

### Initial pH effect

**Figure 8:**
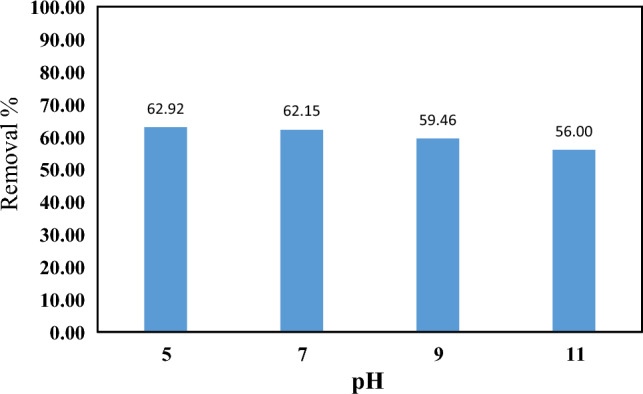
The effect of pH for adsorption of PEN on CMT-g-poly (AAc-*co*-AAm).

The pH of the solution is the first important factor in the adsorption process. To investigate the effect of solution pH on the surface adsorption of PEN on CMT-g-poly (AAc-*co*-AAm), PEN solutions were prepared and their pH was adjusted using sodium hydroxide and hydrochloric acid solutions in desired amounts. Figure 8 shows the results of the study of the effect of pH of solution on the adsorption process of PEN on CMT-g-poly (AAc-*co*-AAm), and as can be seen, there is no significant change in the removal efficiency with changes in pH. In a study conducted by Wang and his colleagues, the removal of triazole compounds was investigated using graphene/Fe_3_O_4_ nanocomposite and it was observed that changes in the pH of the solution have very little effect on the removal efficiency of these compounds^4^. Triazole compounds are usually amphoteric and electrostatic interaction is not effective in the adsorption mechanism of these compounds. It can be said here that interactions related to hydrogen bonding between PEN and CMT-g-poly (AAc-*co*-AAm) are the dominant interactions in the surface adsorption of PEN.

### Adsorption kinetic studies

**Figure 9:**
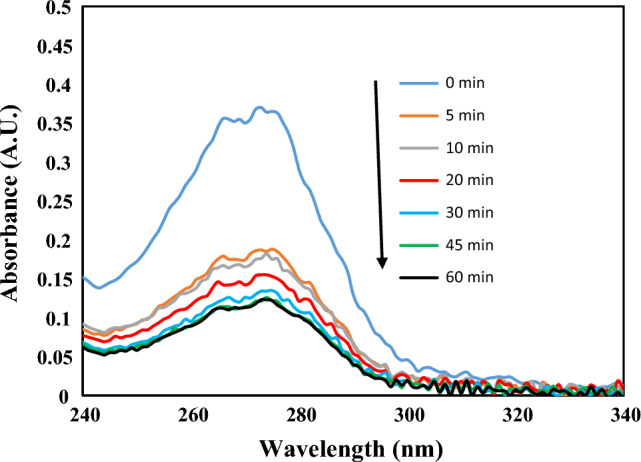
UV–vis absorption spectra of PEN in various times after adsorption onto CMT-g-poly (AAc-*co*-AAm).

When investigating adsorption systems, it is necessary to know the kinetics of the adsorption process because kinetic studies help to determine the amount of pollutant adsorption on the adsorbent surface at a given pressure or concentration. Adsorption kinetics is related to the study of effective factors in reaching equilibrium at a given time. To better understand the adsorption mechanism and the process that controls the adsorption rate, different kinetic models are used. In the present study, adsorption kinetics is investigated using the PFO, PSO, and Elovich models. Figure 9 shows the spectrophotometric spectrum of PEN at different times after contact and surface adsorption on CMT-g-poly (AAc-*co*-AAm). After 45 min of PEN contact with the adsorbent, the adsorption process reaches equilibrium.

**Figure 10:**
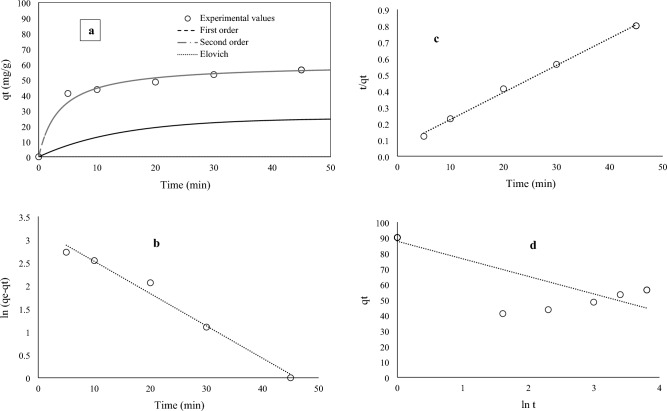
Adsorption kinetic of PEN onto CMT-g-poly (AAc-*co*-AAm) (**a**), the corresponding kinetic plots of pseudo-first order (**b**), pseudo-second order (**c**) and Elovich (**d**) models for the adsorption of PEN onto CMT-g-poly (AAc-*co*-AAm) at initial concentration 30 mg L^−1^.

Adsorption kinetics is usually expressed in four consecutive stages, which include (1) the transfer of the adsorbate from the solution mass to the boundary layer around the adsorbent, (2) the transfer of the adsorbate from the boundary layer to the outer surface of the adsorbent (external penetration), (3) transfer into the adsorbent (internal penetration) and (4) the interaction between the adsorbate and the final sites of adsorption. In the meantime, steps 1 and 4 happen very quickly and are not involved in determining the overall speed of the adsorption process, and external influence and internal influence are the main resistances. Here, the kinetic data of PEN adsorption on CMT-g-poly (AAc-*co*-AAm) were fitted with PFO, PSO, and Elovich kinetic models, as shown in Fig. 10a–d. Also, the values of the constants of these models are presented in Table 1. As can be seen, the pseudo-second-order kinetic model is in better agreement with the kinetic data of PEN adsorption. In this model, the adsorption kinetics is controlled by the absorbent capacity and the contaminant concentration^27^.

**Table 1:** Various kinetic parameters for adsorption of PEN onto CMT-g-poly (AAc-*co*-AAm).

Pseudo-first order		Pseudo-second order		Elovich	
R^2^	0.98	R^2^	0.99	R^2^	0.76
K_1_	0.0700	K_2_	0.00467	β	− 0.08
q_e_	25.19	q_e_	60.24		

### Adsorption isotherm studies

**Figure 11:**
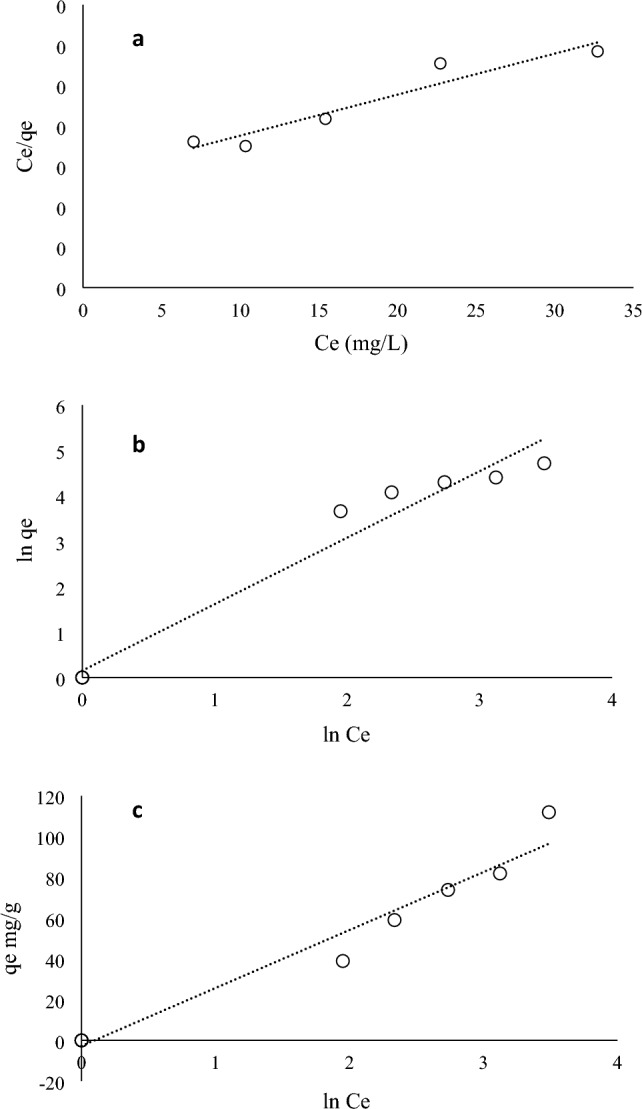
Adsorption isotherms (**a**) Langmuir (**b**) Freundlich and (**c**) Temkin of PEN onto CMT-g-poly (AAc-*co*-AAm).

Adsorption isotherms, which show the relationship between pollutant correlation and adsorption rate, are performed in constant experiments with a series of experiments and compared in adsorption and are very powerful in understanding the adsorption mechanism. Here, the adsorption data obtained for PEN were fitted on three common Langmuir, Freundlich, and Temkin isotherm models to obtain its adsorption mechanism on CMT-g-poly (AAc-*co*-AAm). The amount of adsorption of organic contaminant may be limited due to spatial persistence, but the strength or energy of adsorption depends on the interaction between the adsorbent and the pollutant. Figure 11a–c shows the fitting results of PEN adsorption data on these three models. Table 2 shows the values of the constants of these models along with the correlation coefficients. According to the Langmuir model diagram, the maximum adsorption capacity (q_max_) of the adsorbent for PEN is 196.08 mg/g, which compared to other studies on the removal of PEN, the synthetic hydrogel adsorbent has a higher adsorption capacity^6^. The separation factor (RL) values calculated using the Langmuir isotherm are given in Table 3. According to the correlation coefficients of the regression line, the Freundlich isotherm model is more suitable for describing the surface adsorption of PEN. Also, considering that the Langmuir model is based on the reversibility of the surface adsorption action, and considering the low absorption constant of PEN on CMT-g-poly (AAc-*co*-AAm), it can be said that the Freundlich model is more suitable for describing the process. The Freundlich isotherm model shows the heterogeneity of the contact surface in the surface adsorption process. This model also emphasizes the adsorption of several layers of adsorbent on the adsorbent and the stronger binding sites are occupied by the adsorbent first. Considering that the ratio of 1/n in the Freundlich equation is higher than 1, therefore, the co-adsorption of PEN on CMT-g-poly (AAc-*co*-AAm) is conceivable^23^.

**Table 2:** Various isotherms parameters for adsorption of PEN onto CMT-g-poly (AAc-*co*-AAm).

Langmuir		Freundlich		Temkin	
R^2^	0.912	R^2^	0.965	R^2^	0.962
q_m_	196.08	1/n	1.47	B	28.44
K_L_	0.037	K_F_	1.164	K_T_	0.91

**Table 3:** Values of separation factor (RL) calculated using the Langmuir isotherm.

Concentration (mg L^−1^)	Separation factor (RL)
20	0.57
30	0.47
40	0.40
50	0.35
70	0.28

### Reusability of CMT-g-poly (AAc-*co*-AAm)

**Figure 12:**
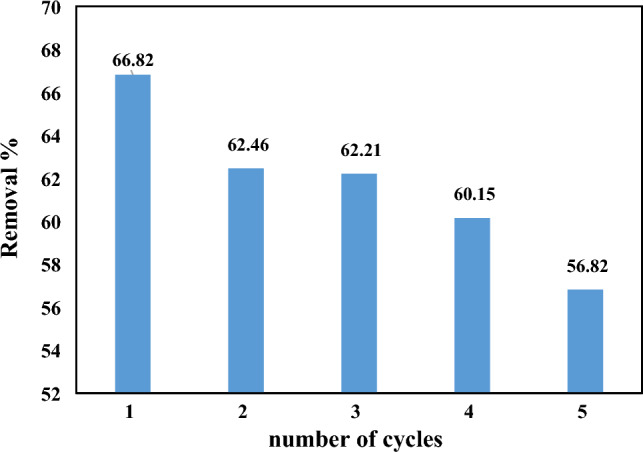
Recycling ability of CMT-g-poly (AAc-*co*-AAm) for PEN removal up to 5 cycles.

To check the reproducibility of CMT-g-poly (AAc-*co*-AAm) in removing PEN, a certain amount of it is placed in contact with the PEN solution and after washing and drying the absorbent, it is placed in fresh solution for the next cycle. It is placed from PEN. In the present study, this work was investigated up to 5 cycles and in the pH range of surface waters. According to Fig. 12, after 5 cycles, the removal rate of PEN reaches 66–56%, which indicates the good stability of CMT-g-poly (AAc-*co*-AAm) in removing PEN.

## Conclusions

In this work, CMT-g-poly (AAc-*co*-AAm) was prepared by the radical polymerization method. Adsorption of the PEN (Topas 20%) on CMT-g-poly (AAc-*co*-AAm) was investigated. The results of the study of the effect of solution pH on the adsorption process of PEN on CMT-g-poly (AAc-*co*-AAm) showed that there is no significant change in the removal efficiency with changes in pH. Triazole compounds are usually amphoteric and electrostatic interaction is not effective in the adsorption mechanism of these compounds. The adsorption isotherm study showed that the results are more consistent with the Freundlich isotherm model. The maximum adsorption capacity obtained from the Langmuir model showed a value of 196.08 mg/g, which is a good value for the synthetic adsorbent. Also, the study of adsorption kinetics shows that the surface adsorption of PEN is better described by the pseudo-second-order model. The reproducibility of CMT-g-poly (AAc-*co*-AAm) in the removal of PEN is favorable for up to 5 cycles. Therefore, according to the good persistence of fungicides in water and the need to reduce and remove them, the synthetic adsorbent can be used as a cost-effective adsorbent to remove these pesticides and have good results.

## Data Availability

The datasets used and/or analyzed during the current study are available from the corresponding author on reasonable request.
